# ICU mortality rates in patients with sepsis before and after the Surviving Sepsis Campaign

**DOI:** 10.1186/cc14095

**Published:** 2015-03-16

**Authors:** J Melville, S Ranjan, P Morgan

**Affiliations:** 1 East Surrey Hospital, Redhill, UK

## Introduction

The aim of this study was to evaluate the effect of the Surviving Sepsis Campaigns on mortality rates, before and after the second surviving sepsis publication, and to assess whether patients with sepsis being admitted to the ICU had a lower APACHE II score on admission. Patients with sepsis, who require ICU care, have an extremely poor prognosis. It has been shown that the mortality rates range from 20.7% (severe sepsis) to 45.7% (septic shock) [1]. The surviving sepsis campaign was initiated in 2002. The first, second and third publications were published in 2004, 2008 and 2012 respectively [2].

## Methods

A retrospective case note review was performed, looking at a sample of 5,954 patients who were 18 years or older who had been admitted to East Surrey Hospital (ESH) ICU between 1 January 2005 and 31 October 2014. The total number of patients with sepsis was 941. We compared results before and after the second publication of the surviving sepsis campaign, looking at mortality rates, age of patients, admission length prior to ICU transfer, APACHE II score and the length of stay on the ICU.

## Results

From the beginning of 2005 to the end of 2008, the mortality rates for septic patients was 51.9% compared with 41.3% from the beginning of 2009 to end of October 2014. Fisher's two-tailed test showed a significant difference (*P *= 0.003) between the mortality before and after the second publication. The median ages before and after 2009 were 63.9 and 64.8 years. The time in hospital before admission to the ICU was greater before 2009 (6.15 days) compared with after 2009 (5.53 days). There was no significant difference (Mann-Whitney test) between the APACHE II scores, with the mean and median score the same at 17.6 and 18 for both groups. The mean length of stay was 1 day longer after 2009 (8.07 days compared with 9.07 days).

## Conclusion

Patients with sepsis admitted to ESH ICU had a 20% relative decrease in mortality after the second publication of surviving sepsis guidelines. The original aim of the campaign was to reduce mortality from sepsis by 25% in 5 years [3]. This decrease was not due to a significant difference between the sets of patients. The decreased time to admittance to ICU may be due to improved recognition of the need for ICU care. Overall the surviving sepsis campaign has had a significantly beneficial effect on mortality rates in patients with sepsis.
